# Hypolipidemic effect of gypenosides in experimentally induced hypercholesterolemic rats

**DOI:** 10.1186/1476-511X-12-154

**Published:** 2013-10-25

**Authors:** Yue-Hui Yang, Jun Yang, Qing-Hua Jiang

**Affiliations:** 1 Pharmaceutical department, Shengjing Hospital, China Medical University, 36 Sanhao Road, Shenyang 110004, China

**Keywords:** Gynosaponins, Hyperlipidemia, Lipid metabolism

## Abstract

**Background:**

To investigate the anti-hyperlipidemic effect of gynosaponins (GPs) in hyperlipidemic rats induced by high lipid diet.

**Methods:**

Animal model of hyperlipidemia was established by high-fat and high-cholesterol diet. Rats were randomly divided into 6 groups, except the normal and model groups, rats in GPs groups were daily administered intragastrically with GPs (50, 100, and 200 mg/kg), and rats in simvastatin group were daily administered intragastrically with simvastatin (10 mg/kg). It was measured that the contents of glutathione peroxidase (GSH-Px), superoxide dismutase (SOD), catalase (CAT), malondialdehyde (MDA), total cholesterol (TC), triglyceride (TG), high-density lipoprotein-cholesterol (HDL-C) and low-density lipoprotein-cholesterol (LDL-C) in the serum, TG and TC in the liver during this experiment, respectively. The left lobe of liver was observed by histopathological staining, and the immunohistochemical staining was used to observe the effects on the effect of GPs on liver functions.

**Results:**

Compared with the model group, GPs groups could remarkably decrease the content of lipids, GSH-Px, SOD, CAT and MDA in the serum and TC and TG in the liver of the hyperlipidemic rats. The pathomorphological results of hepatic tissue showed that fatty degeneration and inflammatory reaction of GPs groups were lightened compared with the model group.

**Conclusions:**

The results show that GPs has good effects on the treatment of hyperlipidemia induced by high lipid diet in rats. The possible anti-hyperlipidemia mechanism maybe those GPs can regulate the disorder of lipid metabolism as well as ameliorate hepatic function.

## Background

Hyperlipidemia has been thought to be a modifiable risk of cardiovascular disease, a most common cause of mortality worldwide, accounting for almost 17 million deaths annually [1]. Generally, bile acid sequestrants, statins, fibrates and nicotinic acids are efficacious antihyperlipidaemic drugs currently available, but adverse reactions and quality of life morbidity indicates the need to find safe and highly effective regimens for dyslipidemia.

**Figure 1:**
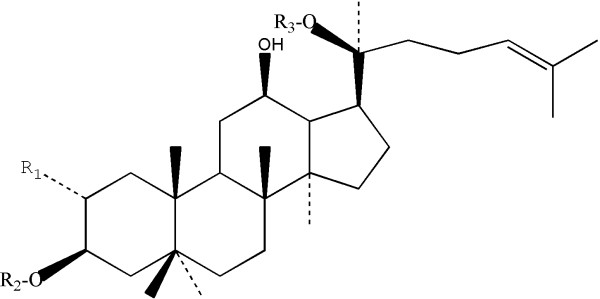
**General structure of dammarane-type gypenosides.** R1/R2=glucose, rhamnose; R3=glucose, xylose.

To date, herbal medicines are widely used to treat variety of disease. Gypenosides (GPs) are triterpenoid saponins contained in an extract from Gynostemma pentaphyllum and are reported to be effective in treating inflammatory [2], cancer and cardiovascular disease [3]. It’s reported that GPs contains 84 saponins, and 4 of them (Gyp-3, 4, 8, 12) are synonym of ginsenoside (Gin-Rb1, Rb3, Rd, F2), including gynosaponins that have been isolated and identified in Asia and suggested to have a variety of pharmacological properties. And 6 of these saponins are the same to those of ginsenosides, such as Gyp-3, 4, 8, 12 are Gin-Rb1, Rb3, Rd, F2 [4]. But the medicinal properties of GPs have been mainly attributed to the saponins. A general structure of dammarane-type gypenoside is illustrated in Figure 1.

Recently, interests in understanding the cardiovascular property of GPs appear to be mounting. And the anti-hyperlipidemic effect has been observed in human and animal studies, with an efficacy that is comparable to most of the current natural products [5,6], suggesting its safety and effective through chronic clinical treatment of hyperlipidemia. However, the mechanism underlying the therapeutic effect is not known. Thus, in this study, we conduct an experiment to investigate the cholesterol-lowering effects of GPs in the high-fat and high-cholesterol induced hyperlipidemic rats.

## Methods

### Materials and agents

Simvastatin (Merk Sharp & Dohme Limited U.K.) was used as a positive control cholesterol-lowering drug. Gypenosides tables were purchased from Hutchison Whampoa Guangzhou Baiyunshan Chinese Medicine Company Limited (Z10900032).

The HFHC rodent chow, containing cholesterol 3%, cholic acid 0.2%, propylthiouracilum 0.5% and lard 10%, was supplied by Guangdong Medicinal Laboratory Animal Center, Guangzhou, China, Certification, SCXK 2008–0002.

### Animal model and treatment

Forty eight adult male healthy Wistar rats (supplied by Guangdong Medicinal Laboratory Animal Center, Guangzhou, China, Certification, GDMLAC 2007A056), weighing 180–200 g, were kept in wire-bottomed cages under a 12 h light/dark cycle and controlled temperature (25 ± 1°C) and relative humidity of 40–60%, and had free access to standard lab chow and tap water. All experimental protocols have got clearance from Institutional Animal Ethics Committee of Guangdong Pharmaceutical University (GDPUIAEC No. 200902), which is in a compliance with national and international guidelines of animal welfare (NIHGuide for the Care and Use of Laboratory Animals, NIH publication No. 85–23, 1985).

After 1 week of accommodation, all of the animals were then divided into 2 groups by weight randomly; group 1, 8 rats were fed with normal diet (ND) until sacrifice, serving as normal-diet controls, while group 2, the other 40 rats were fed with a HFHC diet for continuous 4 weeks to induce hyperlipidemia animal model. Then the rats that fed the HFHC diet after 4 weeks were randomly divided into 5 groups (n=8, respectively) depend on TC levels. GPs table (200 mg/kg, 100 mg/kg and 50 mg/kg) or simvastatin (10 mg/kg) or vehicle in saline was orally administered at the described below dosage respectively, concomitantly continued HFHC diet for 15g/rat/d. The vehicle rats were orally dosed with the same volume of saline, following the HFHC diet for 15g/rat/d. The same subsequent treatments last for continuous 5 weeks. The control rats were administrated with saline and normal lab standard rodent chow instead of HFHC diet and GPs tables for the whole experimental period. During the experiment, animals were weighed weekly, and 24 h food consumption was recorded with the day of treatment with GPs tables or control as day 0.

### Blood sampling

Blood samples were taken (following fasting for 12 hours) before, after, and at the indicated days during the treatment from the eye socket vein of the rats at room temperature for coagulation. Serum samples were obtained by centrifugation at 4000 r/min, 4°C for 10 min. Serum LDL-C, TC, TG, HDL-C, GSH-Px, SOD, CAT and MDA levels were assayed by Automatic Biochemical Analyzer (Aeroset 09D0501, American).

### Liver sampling

Animals were sacrificed by decapitation and peritoneal cavity was flushed immediately with 10 ml of cold saline. Livers were excised and 0.2 g liver of each rat was homogenized in 2 ml precooled chloroform-methanol (1:1) at 4°C to gain a final concentration of 10%. The supernatant was then centrifuged at 4000 r/min, 4°C for 10 min and stored at -70°C for future analysis. The left lobe of liver was cut into 1 cm × 1 cm × 0.5 cm, fixed in 10% formaldehyde, then embedded in paraffin, sectioned, and stained with HE to analysis the histological change by optical microscope.

### Bioassays

Levels of TC, TG, HDL-C and LDL-C, GSH-Px, SOD, CAT and MDA in serum were determined by Automatic Biochemical Analyzer. And the level of TC and TG in the rat liver preparation was determined by Automatic Biochemical Analyzer.

### HMG-CoA Reductase assay

The liver cell pellet was homogenized at 4°C in a Dounce homogenizer in 20 mM PBS (pH 7.4), 1 mM EDTA, and 30 mM nicotinamide. The HMG-CoA reductase assay was performed according to a previous report [7]. The enzyme reaction was linear with time up to 60 min and for protein concentrations up to 0.5 mg. Protein content was measured by the Bradfod method [8].

### Statistical analysis

All results were expressed as mean ± SEM. The data were evaluated by one-way ANOVA, and the differences between the means were assessed using Duncan’s test. *P*< 0.05 was considered as statistically significant.

## Results

### Anti-hyperlipidemic effects of GPs

**Figure 2:**
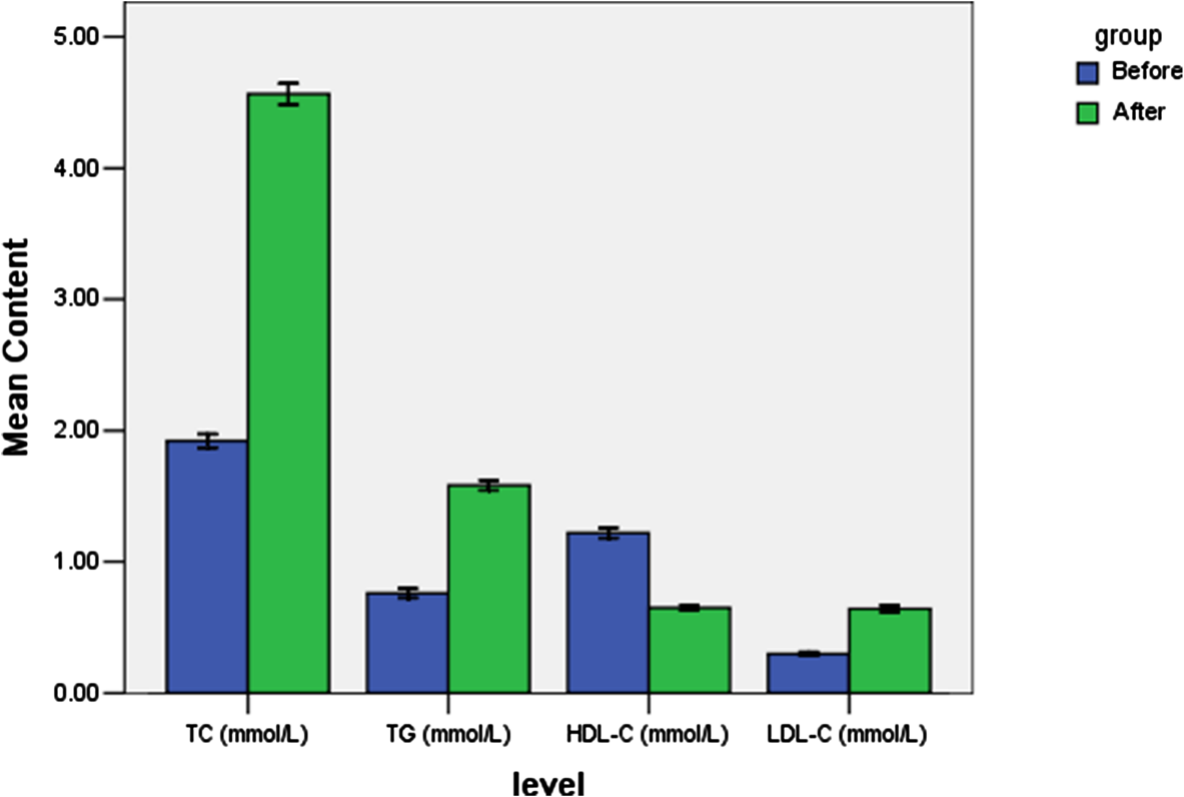
Changes of lipids before and after HFHC-diet for 4 weeks.

As showed in Figure 2, serum TC, TG and LDL-C levels were markedly elevated (from 1.83±0.03 mmol/ml to 4.19±0.11 mmol/ml for TC, 0.74±0.04 mmol/ml to 1.70±0.12 mmol/ml for TG and 0.28±0.01 mmol/ml to 0.70±0.12 mmol/ml for LDL-C), while serum HDL-C levels were significantly decreased (from 1.14±0.03 mmol/ml to 0.67±0.12 mmol/ml) after they were fed with HFHC diet for 4 weeks. And there were statistically significant between the experimentally induced hypercholesteolemic groups and the control group (P<0.05). Administration of GPs table led to significant reduction of serum TC, TG and LDL-C, as compared to those in the hypercholesteolemic model group. In addition, the reduced levels of HDL-C in the HFHC-fed rats were dose-dependently reversed by GPs with a dosage range from 50 to 200 mg/kg.

**Figure 3:**
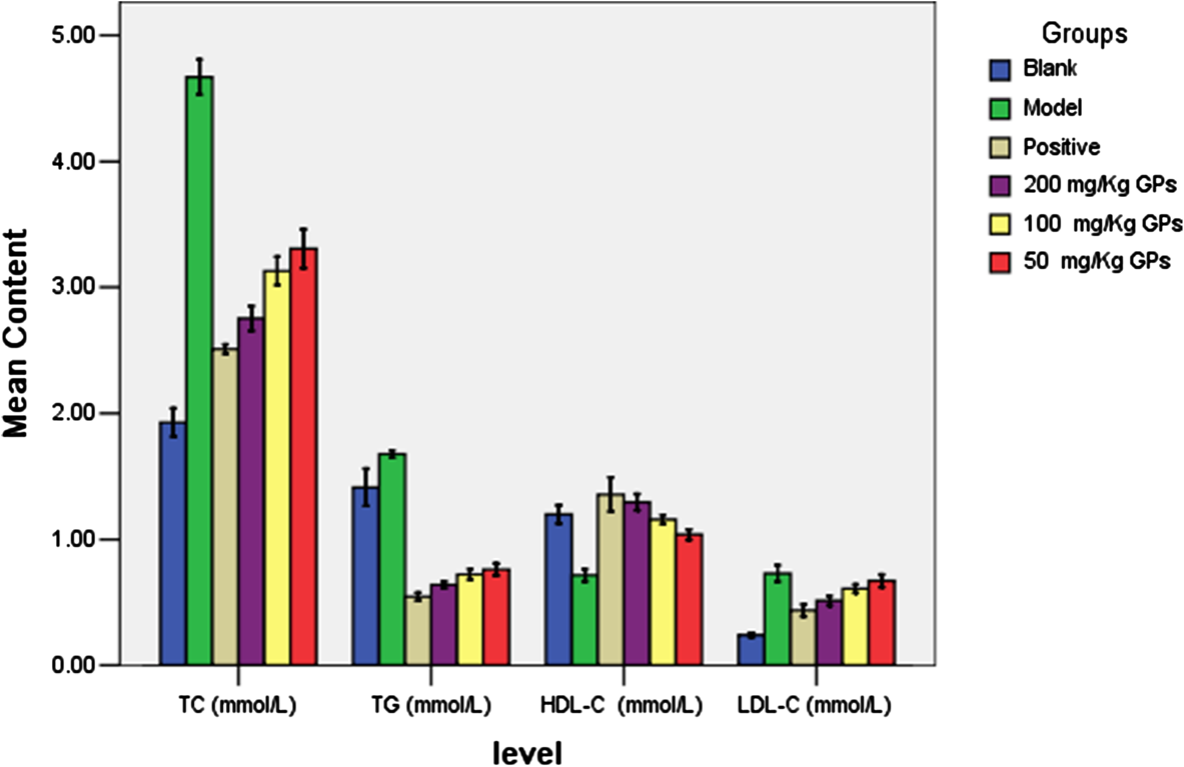
The hypolipdeamic effect of GPs on HFHC-induced hyperlipidemic rats.

In particular, compared with simvastatin (10 mg/kg), GPs tables (200 mg/kg) exhibited a similar effect of the improvement on serum lipid profiles in the hyperlipidemic rats (Figure 3).

### Effects of GPs on serum GSH-Px, SOD, CAT and MDA levels

**Figure 4:**
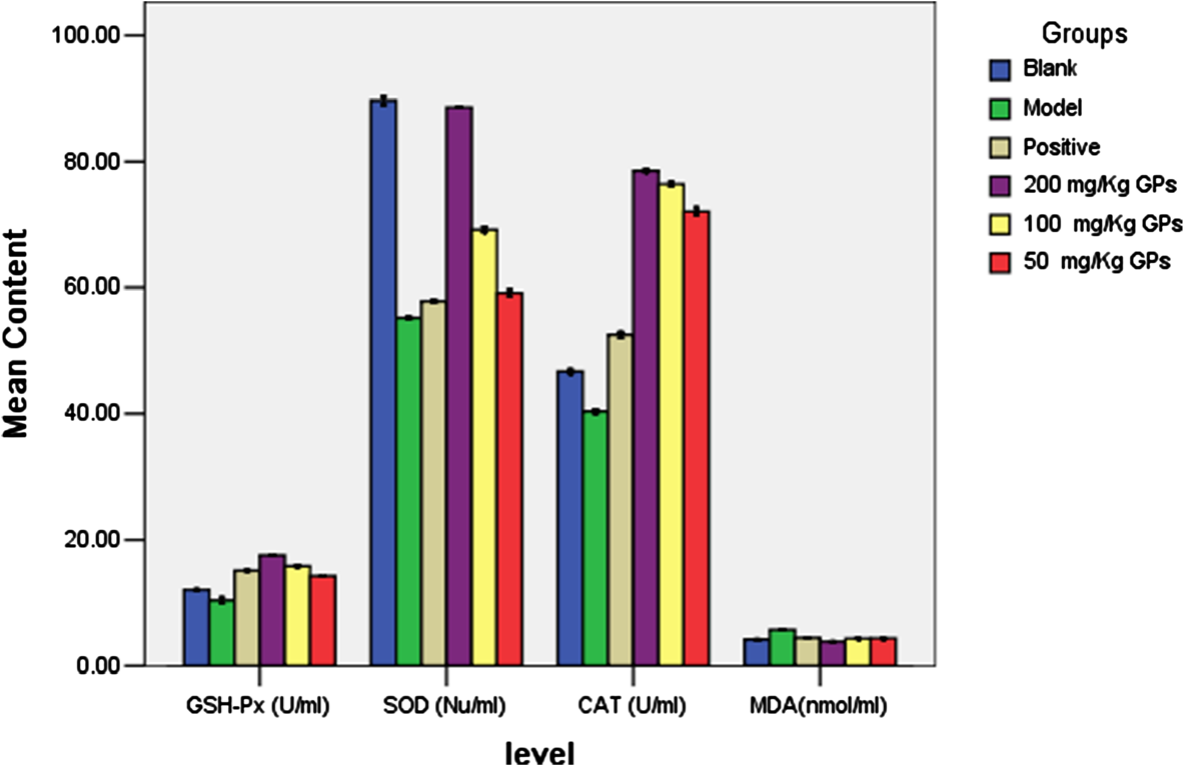
Effects of GPs on hepatic function of HFHC induced hyperlipidemic rats (x±s, n=8 for each group).

Oxidative stress has been regarded as a common pathogenic mechanism in various liver diseases. In Figure 4, the antioxidant activities of GPs were determined by measuring the serum GSH-Px, SOD, CAT and MDA levels. The MDA levels increased in the HFHC model group but decreased levels of SOD and GSH-Px activity compared with those observed in the control group, suggesting that HFHC diet may lead to the imbalance between oxidative stress generation and antioxidant formation. However, GPs treatment significantly reversed those values, indicating that GPs may prevent the pathological process (Figure 4).

### Effects of GPs on TC and TG levels in liver of rats

**Figure 5:**
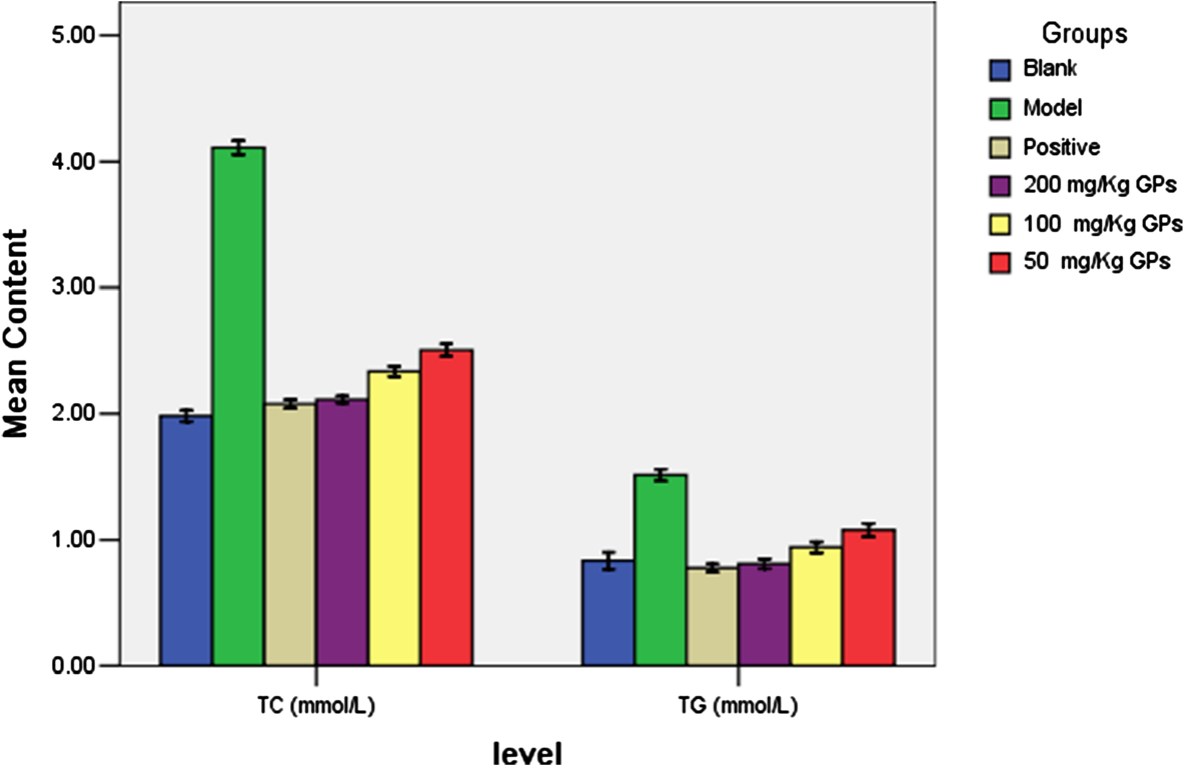
Effects of GPs on TC and TG content in liver of high lipid diet induced hyperlipidemic rats (x±s, n=8 for each group).

As showed in Figure 5, TC and TG levels in liver of HFHC-induced rats were markedly elevated compared with those in the control groups (P<0.05). Administration of GPs table led to significant reduction of liver TC and TG, as compared to those in the hypercholesteolemic model group. In addition, the reduced levels of TC in the HFHC-fed rats were dose-dependently reversed by GPs with a dosage range from 50 to 200 mg/kg.

Importantly, GPs tables (200 mg/kg) showed a similar effect of the down-regulation on liver lipid profiles in the hyperlipidemic rats compared with simvastatin (10 mg/kg), indicating the potential of protecting liver by GPs.

To explore the function of GPs more clearly, the effects of gypenosides on HMG-CoA reductase activity were analyzed here. The result showed that GPs can inhibit the activity of HMG-CoA by 20 to 50% in a dosage-dependent way (Table 1).

**Table 1:** Effects of gypenosides on HMG-CoA reductase activity

		**HMG-CoA reductase activity, pmol mevalonate formed/ mg protein per min**
**Blank group**		50.1 ± 3.2
**GPs group**	50 mg/Kg	40.7 ± 2.8
	100 mg/Kg	32.4 ± 2.2
	200 mg/Kg	24.3 ± 1.9

## Discussion

Dyslipidemia is a metabolic disorder that attributes to atherosclerosis and cardiovascular disease. It is usually elevated serum levels of TC, TG and LDL-C, accompanied by reduced HDL-C levels. According to ATP repots, LDL-C was identified as a powerful atherogenic lipoprotein and was identified as the primary target of cholesterol-lowering therapy. However, HDL-C carries cholesterol and cholesterol esters from the peripheral tissues and cells to the liver, where cholesterol is metabolized into bile acids [9]. It means that increasing HDL-C levels will lower CHD morbidity and mortality. What’s more, dyslipidemia will generate redundant free radicals, such as O^2-^, H_2_O_2_ and ·OH for metabolic disorder. And MDA, a marker of lipid peroxidation and oxidative stress, damages cells and tissues [10].

The current theory of the oxidative modification hypothesis states that LDL becomes oxidized in the arterial wall where it then lends itself to cellular uptake and foam cell formation. It’s said that SOD, CAT and GSH-Px are the three key antioxidant enzymes in the body, which help free radicals to react with other chemicals to produce safe, instead of toxic substances [11].

SOD is responsible for neutralizing the most common free radical, which is known as superoxide. It also aids the body’s utilization of copper, zinc and manganese. Moreover, the SOD enzyme helps protect from gastric damage by converting the highly reactive radicalsuperoxide (O^2-^) into the less reactive peroxide (H_2_O_2_), which can be destroyed by CAT to form water and molecular oxygen. In turn, glutathione-bound enzymes, particularly GSH-Px, the most abundant intracellular nonproteinthiol, have prominent antioxidant and detoxification properties, since they can accept electrons from electron donors, like reduced glutathione, and, thus, form conjugates with some harmful compounds for detoxification. GSH-Px, along with SOD, is one of the body’s endogenous antioxidants and is well known to protect liver cells against oxidative damage through chemical or enzymatic reactions [12-14].

Recently, lots of studies are conducted to investigate the anti-hyperlipidemic mechanism in different models. In the hyperlipidemia rat model induced by P407, gynostemma activity was examined, and it showed to be effective in significantly lowering TG and TC levels, and showed a trend in lowering LDL cholesterol levels in chronic studies. Importantly, they found an improvement of plasma nitrite levels in this model, indicating the potentiality of treating and/or preventing cardiovascular diseases [15]. Furthermore, they also examined the pharmacological anti-hyperlipidemic effectiveness of Gynostemma pentaphyllum in the obese Zucker fatty diabetic rat model. After treatment for 4 days Gynostemma pentaphyllum 250 mg/kg reduced triglyceride (33%), total cholesterol, (13%) and low density lipoprotein cholesterol levels (33%). These effects were dose-dependent and maintained for at least 5 weeks [16,17].

In the study, we investigated the levels of GSH-Px, SOD, CAT, MDA, TC, TG, HDL-C and LDL-C in the serum before/after the treatment and TC, TG in the liver after the treatment. After the treatment for 5 weeks, the levels of TC, TG and LDL-C dose-dependently decreased significantly in the treatment groups compared with the control group. While the HDL-C content was up-regulated in the treatment groups compared with the control group, especially in the dose of 200 mg/kg, which showed a similar effect of the down-regulation on lipid profiles both in the serum and liver in the hyperlipidemic rats compared with simvastatin (10 mg/kg). In addition, compared to the control group, levels of GSH-Px decreased by 13.78%, SOD by 38.41%, CAT by 13.57% while MDA increased by 35.99% in the HFHC-induced group. By contrast, in the simvastatin (10 mg/kg) group, GSH-Px increased by 25.40%, CAT by 12.5%, MDA by 6, but SOD decreased by 35.5%. However, in the dose of 200 mg/kg, levels of GSH-Px increased by 45.57%, CAT by 68.19%, but SOD decreased by 1.18%, MDA by 8.6%. Moreover, in this study, we also observed the marked pathological differences among the six groups. The HFHC-induced hyperlipidemic group showed some pathological abnormalities in liver compared to the control group, which showed normal morphology. The effect of GPs on tissue pathology demonstrated some positive protective findings. We can find some moderate changes in the GPs treatment group compared to the severe changes in the HFHC-induced hyperlipidemic group, investigating the hepatic-protective potential of GPs.

## Conclusion

The results suggest that GPs can effectively regulate the lipid metabolism in the HFHC-induced hyperlipidemic rats and show a hepatic-protective activity. Further research should pay more attention to the hypolipidemic mechanism of GPs.

## Abbreviations

CAT: Catalase; GPs: Gynosaponins; GSH-Px: Peroxidase; HDL-C: High-density lipoprotein-cholesterol; LDL-C: Low-density lipoprotein-cholesterol; MDA: Malondialdehyde; SOD: Superoxide dismutase; TC: Total cholesterol; TG: Triglyceride.

## Competing interests

The authors declare that they have no competing interests.

## Authors’ contributions

YHY performed most of the experiments and prepared the manuscript. YHY, JY carried out the animal studies and biochemical analysis. YHY carried out data collection and analysis. JY helped to draft the manuscript. QHJ participated in the study’s design and coordination. All authors read and approved the final manuscript.

## Authors’ information

Yue-Hui Yang, Jun Yang, and Qing-Hua Jiang: Pharmaceutical department, Shengjing Hospital, China Medical University, 36 Sanhao Road, Shenyang 110004, China.
